# Space-time Analysis of Breast Cancer and Its Late-stage Cases among Iranian Women

**Published:** 2017-10

**Authors:** Meysam OLFATIFAR, Manoochehr KARAMI, Seyed Mehdi HOSSEINI, Masoud PARVIN, Abbas MOGHIMBEIGI, Ahmad KOUSHA, Ali MOTLAGH, Mansoureh ABDOLAHI, Elham PARTOVIPOUR

**Affiliations:** 1. Student Research Committee, Hamadan University of Medical Sciences, Hamadan, Iran; 2. Dept. of Epidemiology, School of Public Health, Hamadan University of Medical Sciences, Hamadan, Iran; 3. Modeling of Noncommunicable Diseases Research Center, Hamadan University of Medical Sciences, Hamadan, Iran; 4. Center for Noncommunicable Disease Control and Prevention, Deputy of Health, Ministry of Health and Medical Education, Tehran, Iran; 5. Cancer Office, Non-Communicable Diseases, Deputy of Health, Ministry of Health and Medical Education, Tehran, Iran; 6. Cancer Research Center, Shahid Beheshti University of Medical Sciences, Tehran, Iran

**Keywords:** Clustering, Spatial analysis, Breast neoplasm, Epidemiology, Iran

## Abstract

**Background:**

Spatial scan statistic has been shown as a useful tool to investigate spatial patterns and detecting the spatial clusters of cancer. This study conducted to study spatial analysis of breast cancer and its late-stage cases, one of the most common women cancers in Iran and the world.

**Methods:**

We used space-time and purely spatial scan statistic implemented in SaTScan software to detect clusters of breast cancer and late-stage cases, at city level by applying Poisson and Bernoulli distribution. Data on 40017 of breast cancer cases that reported to the Ministry of Health and Medical Education (MOHME) during 2005 to 2010 were included.

**Results:**

Purely spatial and spatiotemporal high rates significant clusters of breast cancer and its late-stage cases with Poisson distribution were in the same geographical area including southwest, north, and northeast.

**Conclusion:**

Significant clusters areas have probably differences with other areas in terms of delay in diagnosis and access to appropriate health services because late-stage breast cancer cases had the greatest impact on formation of clusters. However, more studies are essential to be conducted in different areas of country to explain more precisely clusters detected areas and detecting reasonable justification for existence of significant clusters.

## Introduction

Breast cancer with more than one million cases yearly is the most common cancer in women and is responsible for one-fifth of women cancers worldwide (1). Industrialized sectors, of the world, have experienced rapid increase in the incidence of breast cancer in the last decade and still have high incidence rates (2). Estimated breast cancer incidence and mortality age-standardized rates in Iran for 2012 were 24.1 to 33.9 and less than 10.1 per 100000 people respectively (3). The breast cancer diagnosis is the main determinant of patient’s complication (4). Therefore, early detection is the most basic way to control of cancer as the late-stages of it have low survival (5) and have further deterioration.

Surveillance of routinely collected data for unusual clusters of disease in time and space is a key issue (6) and cluster investigations are essential part of responding to community concerns even if the etiologic information not achieved (7, 8). So that detecting the spatial pattern of cancer could be incentive for further investigation and prioritize of the health resources for prevention and treatment in different geographical areas(9).

Scan statistic developed by Kulldorff has been shown as a useful tool to study geographical patterns and detect the spatial clusters of cancer (9). However, although the few studies have been conducted in Iran to investigate of breast cancer distribution, a study has not been conducted in order to space-time clustering of breast cancer cases at province or city level.

Thus, the aim of this study was to detect clusters with high and low rates of breast cancer and its late-stage cases using discrete Poisson distribution and finally investigate spatial distribution of the late-stage breast cancer cases with Bernoulli distribution. Since, the last-grade cases known as a good proxy of screening performance and access to health services.

## Methods

All data of 40017 women diagnosed with breast cancer between 2005 and 2010 were obtained from the Ministry of Health and Medical Education (MOHME). Available data for each case was age, province, city of residence, date of diagnosis, and grade of cancer. City location was selected for analysis as aggregation unit. However, 10.9% of the cases (4396 cases) despite provincial information had not been assigned to a particular city.

Therefore, mentioned cases in each province were distributed randomly throughout city by proportion of women population in each city. SaTScan software ver.9.3.1(10) spatial scan statistic was used to performing space-time and purely spatial analysis. In summary, in this method software impose a search window into map, which in turn placed on the coordinate center of each block and with calculating the likelihood ratio and comparing with amount calculated based on Monte Carlo simulation test the null hypothesis. Discrete Poisson distribution was used to explore the purely spatial and spatiotemporal clusters with high and low rates of breast cancer and was adjusted for age and grade of cancer, the likelihood ratio calculates based on following formula:

$${\left(\frac{c}{\mathrm{EI}/\left(c\right)}\right)}^{c}\;{\left(\frac{C-c}{C-E\left(c\right)}\right)}^{C-c}$$

Where C is the total number of cases, c is the observed number of cases within the window and E[c] is the covariate-adjusted expected number of cases within the window under the null-hypothesis. Since the analysis is conditioned on the total number of cases observed, C-E[c] is the expected number of cases outside the window.

As well to explore the spatial and spatiotemporal clusters with high and low rates of late-stage breast cancer cases Bernoulli distribution was used (late-stage cases=case and other = control) in this case the likelihood ratio calculated based on following formula:

$${\left(\frac{c}{n}\right)}^{c}\;{\left(\frac{n-c}{n}\right)}^{n-c}\;\left(\frac{C-c}{N-n}\right){\left(\frac{\left(N-n\right)-\left(C-c\right)}{N-n}\right)}^{\left(N-n\right)-\left(C-c\right)}$$

Where c and C are defined as above, n is the total number of cases and controls within the window, while N is the combined total number of cases and controls in the data set.

Then, to understand better of the late-stage cases distribution 65.31 percent of breast cancer cases (26136 cases) discrete Poisson distribution was used and analysis was adjusted for age. In order to build files needed for methods, cancer registry data and population data of Iran's cities was used. Population data were obtained from the Statistical Center of Iran (11), for the 2006 and 2011 Census. Geographic information system (GIS) was used to determine the coordinate’s center of cities to build coordinate file and draw the maps, as well. We considered the *P* value less than 0.05(α) as statistically significant. The simulation process was run 999 times.

## Results

### Discrete Poisson model

#### Purely spatial clusters

**Fig. 1:**
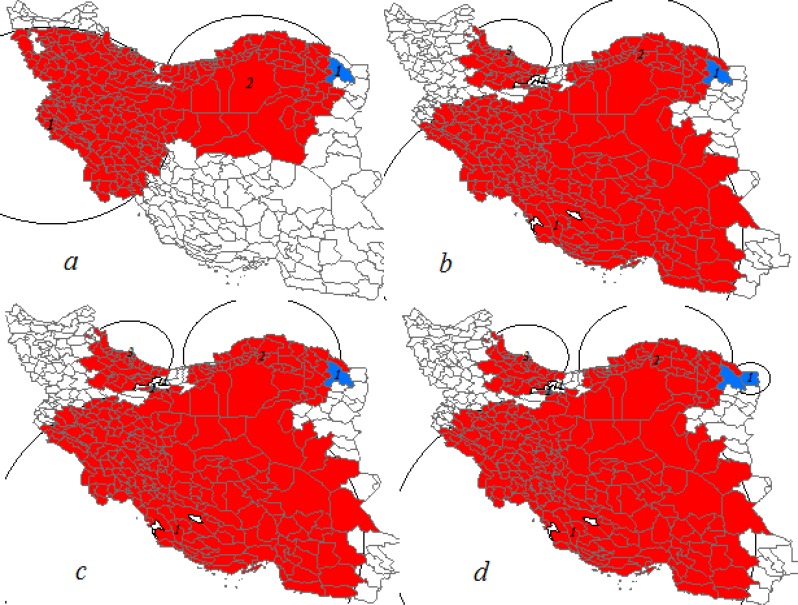
Breast cancer and its late-stage cases clusters at city level in Iranian women for years 2005–2010 Poisson distribution; (a) low and high rates purely spatial clusters of breast cancer cases adjusted for age and grade; (b) low and high rates space-time clusters of breast cancer cases adjusted for age and grade; (c) low and high rates purely spatial clusters of late-stage breast cancer cases adjusted for age; (d) low and high rates space-time clusters of late-stage breast cancer cases adjusted for age (reds and blues color areas are respectively high and low rates clusters and whites color areas aren’t significant)

Spatial scan statistic with discrete Poisson distribution adjusted for age and grade of cancer detected three primary purely spatial clusters of breast cancer in study areas (Table 1). Tow purely spatial clusters with high rates composed of 180 and 73 cities respectively (Fig. 1a). In addition, detected cluster with low rates composed of a city (Fig. 1a) clusters with high and low rates have located in northwestern, west and northeastern of Iran. The first primary cluster had the highest log likelihood ratio. Another characteristic of detected clusters showed in Table 1.

**Table 1:** Significant primary clusters of breast cancer cases with higher and lower rates using discrete Poisson adjusted for age and grade

***Clusters ID***	***Center coordinates***	***Radius (km)***	***No. of cases***	***Exp*^*a*^**	***RR*^*b*^**	***LLR*^*c*^**	***P***
Purely spatial							
High-rates Most likely cluster							
1	33.644N–46.187E	534.93	15573	4445.99	5.10	10351.438	0.001
2	35.631N–55.682E	377.02	3969	551.54	7.88	4568.006	0.001
Low-rates Most likely cluster							
1	36.250N–59.814E	0	2353	2886.43	0.80	56.456	0.001
Space-time							
High-rates Most likely cluster							
1	27.937N–52.237E	865.34	10909	3391.89	4.05	6057.266	0.001
2	37.151N–56.236E	328.72	1433	179.33	8.25	1744.485	0.001
3	37.330N–49.986E	180.38	1494	266.16	5.79	1368.664	0.001
4	35.917N–51.610E	0	29	0.41	70.08	94.644	0.001
Low-rates Most likely cluster							
1	36.250N–59.814E	0	1019	1305.49	0.77	35.082	0.0046
2	35.610N–51.030E	0	0	6.84	0	6.843	0.664

a expected number of cases in clusters

b relative risk of the clusters

c log likelihood ratio

Poisson distribution of the late-stage breast cancer cases adjusted for age detected five primary purely spatial clusters with high and low rates in study areas (Table 2). Clusters with high rates composed of 202, 48, 36, and 1 city respectively (Fig. 1b).

**Table 2:** Significant primary clusters of late-stage breast cancer cases with higher and lower rates using discrete Poisson distribution adjusted for age

***Clusters ID***	***Center coordinates***	***Radius (km)***	***No.of cases***	***Exp*^*a*^**	***RR*^*b*^**	***LLR*^*c*^**	***P***
Purely spatial							
High-rates Most likely cluster							
1	27.937N–52.237E	865.34	11692	3678.02	4.94	7147.142	0.001
2	37.151N–56.236E	328.72	1506	188.85	8.40	1843.709	0.001
3	37.330N–49.986E	180.38	1685	305.54	5.83	1535.095	0.001
4	35.917N–51.610E	0	29	0.44	66.26	93.034	0.001
Low-rates Most likely cluster							
1	36.250N–59.814E	0	1464	1740.76	0.83	24.83	0.042
Space-time							
High-rates Most likely cluster							
1	27.937N–52.237E	865.34	10499	3169.98	4.86	6562.697	0.001
2	37.151N–56.236E	328.72	1380	166.28	8.71	1735.394	0.001
3	37.330N–49.986E	180.38	1432	249.67	6.01	1346.372	0.001
4	35.917N–51.610E	0	29	0.41	70.11	94.648	0.001
Low-rates Most likely cluster							
1	36.291N–60.781E	86.73	78	146.71	0.53	19.523	0.083
2	35.610N–51.030E	0	0	5.76	0	5.763	0.857

a expected number of cases in clusters

b relative risk of the clusters

c log likelihood ratio

In addition, detected cluster with low rates had composed of a city (Fig. 1b). Clusters with high and low rates had been located in southwestern, northern, northeastern areas and somewhat southern areas of country (Fig. 1b).

The center coordinates, radius, number of the observed count, expected count, relative risk, log likelihood, and p-value for each cluster are shown in Table 2.

#### Space-time clusters

The space-time scan statistic detected six primary clusters with high and low rates of breast cancer in study areas (Table 1). Clusters with high rates had composed of 202, 48, 36 and 1 city respectively (Fig. 1c). Three primary clusters with high rates were located in duration of 2008–2010 and fourth cluster was located between 2009 and 2010. Each of two low rates clusters had composed of one city (Fig. 1c). Time of the first cluster was between 2005 and 2010 and for the second cluster was 2008. Location of the high rates cluster in this case and high rates purely spatial clusters of late-stage cases were same as well as location of the first low rates cluster was like with the first purely spatial low rates cluster of late-stage breast cancer cases (Fig. 1b,c). Clusters with high and low rates had been located in areas of southwestern, northern, northeastern and somewhat southern areas of Iran (Fig. 1c). The first primary cluster had the highest log likelihood ratio and radius Table 1 and Fig. 1b.

Poisson distribution of last-stage breast cancer adjusted for age detected six primary clusters with high and low rates in the study areas (Table 2). Clusters with high rates composed of 202, 48, 36, and 1 city respectively (Fig. 1d). Three Primary clusters with high rates were located during 2008–2010 and the fourth cluster was located in 2009–2010. Two low rates cluster had consisted of one and two cities respectively (Fig. 1d). The first was located in 2007 and second was located during 2007–2008. Clusters with high and low rates had been located in areas of southwestern, northern, northeastern and somewhat southern areas of Iran (Fig. 1d). Places of detected clusters in this case were similar to location spatiotemporal clusters of breast cancer and high rates purely spatial clusters of late-stage cases detected by Poisson distribution even time period of high rates space-time clusters were exactly same however the first low rates cluster, in this case, had consisted of two cities (Fig. 1d,b,c). Characteristic of detected clusters showed in Table 2.

### Bernoulli model

#### Purely spatial

**Fig. 2:**
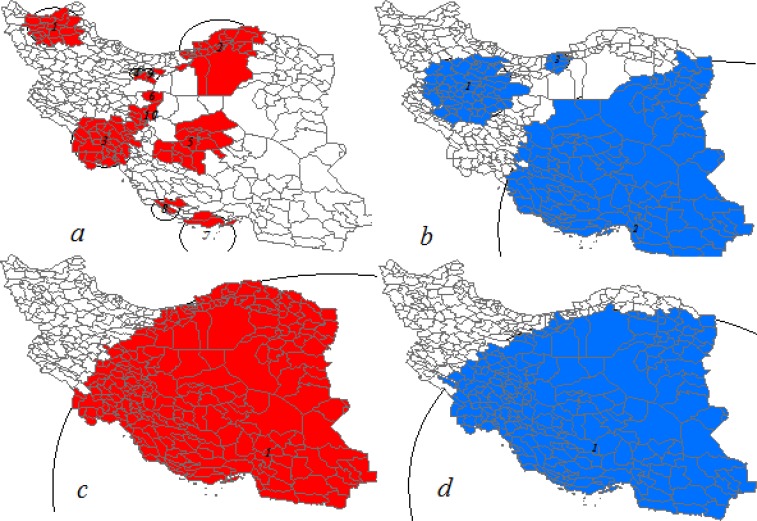
Late-stage Breast cancer cases clusters at city level in Iranian women for years 2005–2010 using Bernoulli distribution; (a), high rates purely spatial clusters; (b) low rates purely spatial clusters; (c) high rates space-time clusters; (d) low rates space-time clusters [whites color areas are not significant]

Spatial scan statistic with Bernoulli distribution detected 15 purely spatial clusters with high and low rates of late-stage breast cancer cases in study areas (Table 3). Purely spatial clusters with high rates had composed of 21, 25, 34, 7, 14, 2, 5, 5, 4 and 9 cities respectively (Fig. 2a). Moreover, three Primary clusters were detected whit low rates; each had consisted of 55, 122 and 8 city respectively (Fig. 2 b). Clusters with high and low rates were located in different areas of the country and along north-south axis (Fig. 2a, b).The first primary cluster had the highest log likelihood ratio but second of theme had the highest radius Table 3.

**Table 3:** Significant primary clusters of late-stage breast cancer cases with higher and lower rates using Bernoulli distribution

***Clusters ID***	***Center coordinates***	***Radius (km)***	***No. of cases***	***Exp*^*a*^**	***RR*^*b*^**	***LLR*^*c*^**	***P***
Purely spatial							
High-rates Most likely cluster							
1	38.227N–46.823E	123.84	1407	1295.55	1.09	29.507	0.016
2	36.988N–55.340E	183.37	745	673.75	1.11	23.544	0.038
3	31.533N–49.406E	164.24	2346	2236.37	1.05	16.147	0.036
4	35.501N–51.071E	35.92	879	821.49	1.07	11.947	0.017
5	31.454N–53.814E	156.25	630	595.01	1.06	5.972	0.351
6	34.171N–51.872E	0	27	21.92	1.23	5.633	0.457
7	26.023N–54.702E	142.26	66	57.63	1.15	4.034	0.927
8	27.570N–52.517E	69.62	30	25.16	1.19	3.511	0.988
9	35.450N–51.778E	40.45	373	352.30	1.06	3.507	0.988
10	33.020N–51.753E	84.56	638	612.06	1.04	3.125	0.997
Low-rates Most likely cluster							
1	34.824N–48.799E	206.83	3942	4117.20	0.95	21.697	0.023
2	26.458N–61.375E	1099.11	9347	9506.40	0.98	9.745	0.016
3	36.350N–53.347E	52.82	560	588.52	0.95	3.558	0.966
Space-time							
High-rates Most likely cluster							
1	25.606N–61.164E	1446.72	12203	10264.58	1.29	1754.927	0.001
Low-rates Most likely cluster							
1	26.490N–57.229E	1159.79	4789	6716.42	0.67	1581.993	0.001

a expected number of cases in clusters

b relative risk of the clusters

c log likelihood ratio

#### Space-time clusters

Space-time scan statistic whit Bernoulli distribution detected two clusters with high and low rates of late-stage breast cancer cases in study areas (Table 3). High rates cluster had composed of 234 city clusters (Fig. 2c) and its time was during 2008–2010, as well as cluster with low rates had composed of 215 cities during 2005–2007 (Fig. 2d). Cluster with high and low rates, except for areas located in the northwest of the country had covered other areas (Fig. 2 c,d). Characteristic of detected clusters showed in Table 3.

## Discussion

We found several significant clusters for both breast cancer and its last-stage cases in study areas. Poisson model in total detected 18 primary space-time and purely spatial significant clusters with high and low rates of breast cancer and its late-stage cases. Space-time and purely spatial clusters with high rates of last-stage breast cancer cases were similar with high rates space-time clusters of breast cancer cases (Fig. 1d,b,c). However, relative risk, estimated number of cases and likelihood ratio of clusters were different (Table 1, 2). Bernoulli distribution detected 10 high and low rates primary significant cluster of late-stage breast cancer cases from total 15 detected clusters in study areas. The existence of such clusters in study areas can represent similarity of special features in these areas. One other hand location similarity of detected clusters with Poisson distribution for breast cancer cases and its late-stage cases may indicate the importance of late-stage cases and more influence of these cases in formation of clusters. Therefore, more attention to these areas is essential in terms of delay in diagnosis and access to appropriate health services, since late-stage breast cancer cases are an appropriate proxy for screening effectiveness and access to health services. Clearly and correctly of detected clusters areas could not be trusted regardless attention to socio-economic affecting factors, race, and ethnicity, location of living and accessibility to appropriate health services.

In order to detect the late-stage breast cancer cases used discrete Poisson distribution instead of the Bernoulli distribution, which does not allow covariates adjustment (12). Poisson distribution is a very good estimator of the Bernoulli distribution, results of this study indicated that areas of detected clusters changed after adjustment for influencing variables. Spatial distribution of detected clusters areas of breast cancer mortality was changed after covariates adjustment (9).

The relationship of late-stage breast cancer diagnosis was studied with access to health services in Illinois and showed that less access to health services associated with later diagnosis of breast cancer (4). Although, there is no published study in Iran to detect breast cancer significant clusters using spatial scan statistic. However, the relationship of breast cancer with socio-economic factors, ethnicity or other differences factors has been studied with other methods. In a study to compare the breast cancer patterns in public and private health sectors showed that socioeconomic differences among different levels of society require more attention to reduce disease, treatment and prevent delay in diagnosis (13). The relationship of breast cancer was studied with ethnic differences in Golestan province and showed that more studies are needed to understand clearly of ethnicity differences (14), and finally positively relationship of breast cancer diagnosis grade was showed with socio-economic level (15). Therefore, studies carried out in different areas of Iran have shown the relationship between breast cancer and its late-stage cases with influential factors.

One of the main limitations of our study was lack of access to socio-economic factors, areas differences in terms of access to appropriate health services and other affecting factors that may affect our findings. Another limitation was continuous changes in number of city in some province so that over years of study we have had changes in more than ten provinces therefor was need to carefully study of any changes in order to true assignment of cases to provinces cites. Despite current study is the first national survey on breast cancer and its late-stage cases using spatial scan statistic.

## Conclusion

Significant clusters areas have different from other areas in terms of delay in diagnosis and health services coverage because late-stage breast cancer cases had the greatest impact on formation of clusters; however, more studies are essential to be conducted in different areas of country to explain more precisely clusters detected areas and detecting reasonable justification for existence of significant clusters.

## Ethical considerations

Ethical issues (Including plagiarism, informed consent, misconduct, data fabrication and/or falsification, double publication and/or submission, redundancy, etc.) have been completely observed by the authors.
